# Strengthening HIV Activism Among Clinicians in Malaysia: A Randomised Controlled Trial

**DOI:** 10.1007/s10461-025-04829-1

**Published:** 2025-07-21

**Authors:** Norman Chong, Iskandar Azwa, Asfarina Amir Hassan, Mohammad Mousavi, Pui Li Wong, Rong Xiang Ng, Rumana Saifi, Sazali Basri, Sharifah Faridah Syed Omar, Suzan M. Walters, Zachary K. Collier, Marwan S. Haddad, Frederick L. Altice, Adeeba Kamarulzaman, Valerie A. Earnshaw

**Affiliations:** 1 Centre of Excellence for Research in Infectious Diseases and AIDS (CERIA), Universiti Malaya, Kuala Lumpur, Malaysia; 2 Department of Medicine, Faculty of Medicine, Universiti Malaya, Kuala Lumpur, Malaysia; 3 Department of Human Development and Family Sciences, University of Delaware, Newark, USA; 4 Neag School of Education, University of Connecticut, Connecticut, USA; 5 Department of Population Health, New York University, Grossman School of Medicine, New York, USA; 6 Center for Drug Use and HIV/HCV Research, New York, USA; 7 Center for Key Populations, Community Health Center, Inc., Middletown Connecticut, USA; 8 Yale School of Public Health, Yale University, New Haven, USA; 9 Yale School of Medicine, Department of Internal Medicine, Yale University, New Haven, USA; 10 Kirby Institute, University of New South Wales (UNSW) Sydney, Sydney, Australia; 11 Monash University Malaysia, Subang Jaya, Malaysia; 12 Department of Social and Preventive Medicine, Faculty of Medicine, Universiti Malaya, Kuala Lumpur, Malaysia; 13 Department of Pharmaceutical Chemistry, Faculty of Pharmacy, Universiti Malaya, Kuala Lumpur, Malaysia

**Keywords:** HIV, Activism, Key Populations, People with HIV, Project ECHO, Stigma, Physicians

## Abstract

HIV continues to disproportionately affect key populations in Malaysia, compared to the general population. Lessons learned from decades of research and programmatic experience suggest that HIV activism can be a driver for change. We pilot-tested a tele-training platform, Project ECHO^®^ for Stigma Reduction (PE-SR), in a randomised controlled trial from July 2022 to March 2023, alongside two comparator groups, i.e., Project ECHO^®^-Standard (PE-S) and the conventional HIV training program for clinicians, HIV Connect (HC). We randomised 78 primary care physicians and general practitioners across Malaysia into the three study arms (*n* = 26 each). We evaluated changes in HIV activist identity and commitment, and orientation towards day-to-day HIV activism and structural HIV activism. Repeated measure analysis of covariance (ANCOVA), controlling for age, years of practice, and contact with key populations as covariates, compared changes in HIV activism constructs across time and groups. The randomised controlled trial yielded mixed results. We observed statistically significant changes in HIV activist identity and commitment, as well as changes in orientation towards structural activism in all groups. We also found statistically significant mean differences between PE-S and HC in terms of HIV activist identity and commitment, and between PE-SR and HC in terms of orientation towards structural activism. Results suggest that stigma reduction tools embedded in a tele-training platform had a preliminary impact on HIV activism and could be scaled up and tailored to train clinician-activists.

*Trial Registration* NCT05597787.

**Supplementary Information:**

The online version contains supplementary material available at 10.1007/s10461-025-04829-1.

## Introduction

The successes in the global HIV response have a long-standing history in HIV activism led by people with and affected by HIV, particularly in advancing political action and dismantling stigma. Yet, in resource-limited settings such as Malaysia, the HIV prevalence continues to disproportionately affect key populations, underscoring the urgency of addressing widening health inequities [1, 2]. Stigma, particularly from clinicians, remains an impediment to achieving favourable strides in the HIV response in Malaysia to scale up testing modalities, preventive strategies, and efforts to strengthen engagement in care [1, 2, 3, 4]. Clinicians occupy a pivotal position in patient care [5] and are likely to be regarded as influential or key opinion leaders within and beyond healthcare institutions [6, 7]. Clinicians bear the ethical responsibility to meet the health needs of the individuals under their care, including supporting structural and sociopolitical reforms [8]. However, healthcare professionals may perceive involvement in addressing sociopolitical determinants of health as beyond their area of influence and expertise [9]. The hierarchical structure of health systems [10] and concerns about potential repercussions towards employment security may also dissuade clinicians from practising activism [11]. Additionally, many medical schools and residency curricula lack training in social justice, allyship, and activism [12]. We address this gap by evaluating a pilot intervention to improve HIV activism among clinicians in Malaysia via Project ECHO^®^ (Extension for Community Healthcare Outcomes), a healthcare decentralisation tele-mentoring program that trains and supports non-specialty clinicians to provide specialty care.

HIV activism spans efforts and initiatives directed towards achieving societal improvement in the HIV response by promoting healthcare, economic, and sociopolitical justice for individuals with and affected by HIV/AIDS. These efforts can include attending planning committee meetings, contesting stigmatising statements, product boycotts, and peaceful protests [13, 14, 15]. Aderson and Eskandari [16] introduced a multidimensional conceptualization of activism from a feminist perspective, in that it extends across time (evolving pre-, during, and post-conflict), space (occurring in diverse settings, such as homes, communal areas, or institutions), scale (addressing disparities at various levels, from local to international) and type (challenging structural, cultural or symbolic forms of oppression). In employing social movement theory within health social movements, Brown and colleagues [17, 18] outlined three main objectives of health activism: (1) Health access movements, to promote equitable access to healthcare services through national healthcare reforms and the expansion of health insurance coverage, (2) person-centred initiatives, aimed at leveraging lived experiences of illness and disability in support of scientific evidence, and (3) community-centric health movements, which prioritizes constituencies affected by health disparities. Empowerment-based interventions are developed to include activism, solidarity, resilience, and social support [19]. On the structural level, activism is a powerful tool for improving sociopolitical will, changing cultural norms, and stigmatising policies [20].

HIV activism has also contributed to the progress in the HIV response in Malaysia. Because injecting drug use was the early driver of the HIV epidemic in Malaysia, much of the earlier HIV activism focused on decriminalisation of drug use and possession alongside the provision of harm reduction services such as the needle-syringe exchange program and opioid agonist therapy [21]. The Ministry of Health subsequently endorsed harm reduction as part of the national healthcare services. Over the course of the epidemic, the HIV prevalence among PWID reduced significantly, and the harm reduction services demonstrated a compelling return on investment [22]. In 2003, Malaysia authorised compulsory licensing for antiretroviral medications, making generic drugs more accessible. Besides reducing the purchasing cost of these medications by 81%, it also doubled treatment capacity [23]. Most recently, the abolition of the mandatory death penalty bill [24] became a stepping stone towards the decriminalisation of drug possession. Additionally, the dispensing of free-of-charge HIV pre-exposure prophylaxis (PrEP) at public health clinics is en route to be fully endorsed and subsidised by the Ministry of Health for a nationwide PrEP provision in all primary care clinics following strong preliminary effectiveness [25].

### Current Study

Project ECHO^®^ is a high-impact virtual and synchronous tele-mentoring network to facilitate knowledge transfer from speciality experts (termed as ‘hub members’) to non-speciality clinicians and leverage collaborative learning to empower clinicians to provide speciality care while retaining care responsibility in their respective communities. The Project ECHO^®^’s hub-and-spoke model mimics routine medical rounds through knowledge sharing, clinical case learning, and learning loops. Project ECHO^®^ demonstrated improvements in patient-centred integrated care in chronic health conditions, including HIV treatment and preventive strategies, LGBT health, and non-communicable diseases [26, 27, 28]. Project ECHO^®^ has a network of 896 hubs across 63 countries, reaching over four million clinicians [29].

The current study is a part of a larger parent study that evaluates a pilot intervention to reduce stigma among primary care physicians and general practitioners in Malaysia through the Project ECHO^®^ tele-mentoring platform. We anticipate that an intervention with evidence-based tools to reduce stigma (i.e., education, contact facilitation between potential stigmatisers and stigmatised, participatory learning and social norming) could improve intention to engage in HIV activism and elicit behavioural changes in HIV activism. As part of the main study, this study evaluates the impact of the intervention on improving HIV activism. Informed by the disparities in the HIV response in Malaysia [30, 31], structural stigma [32, 33, 34, 35] and reported HIV-related stigma among clinicians and medical students [1, 2, 3, 4, 36], the intervention identified the potential of general practitioners and primary care physicians in advancing the HIV response as clinician-activists. In a randomised controlled trial, the aims of the study were to evaluate the impact of the intervention on HIV activism and to identify who among the clinicians is more likely to identify as HIV clinician-activists and engage in HIV activism.

## Methods

### Study Design

The intervention ran as a single-blinded randomised controlled trial, wherein recruited individual clinicians were randomised to intervention arms (see Appendix 1 for CONSORT flow chart).

We calculated the sample size in R using the power package for single-level generalised linear models based on recommendations from Cohen’s A Power Primer [37] and Kadam and Bhalerao [38]. The study aimed to achieve 80% power to detect a statistically significant (*p* < 0.05), medium effect size (Cohen’s d = 0.5), type I error (α) of 5%, confidence interval of 95%, and standard deviations (SD) of 0.78, based on preliminary findings of stigma among Malaysian clinicians [1, 36] and future healthcare providers [2, 4]. The power analyses recommended the recruitment of 64 participants for this study. We recruited an additional 14 participants (22% of the sample size) to account for potential attrition. Thus, this study recruited 78 participants who subsequently provided consent.

Participants (*n* = 78) were randomised into three study arms (*n* = 26 in each arm). The arms are HIV Connect (HC), Project ECHO^®^: Standard (PE-S), and Project ECHO^®^: Stigma Reduction (PE-SR). Both Project ECHO^®^ arms were titled Project ECHO^®^ for HIV Prevention to ensure participants were blinded to their assigned study arms and their content. The clinicians’ allocation sequence to intervention arms was also concealed from the hub members who delivered the intervention. The clinicians received didactic lectures in HIV testing, prevention, and linkage to care across every condition based on guidelines from the Malaysian Ministry of Health, the Center for Disease Control and Prevention, and the World Health Organisation. All three study arms ran over the course of nine months.

#### HIV Connect (HC)

This served as the treatment-as-usual comparator. Participants received the HC modules from the Malaysian Society of HIV Medicine (MASHM), dedicated to informing Malaysian primary care physicians about HIV. The modules led by HIV infectious disease experts covered essential topics, including epidemiology, pre-exposure prophylaxis (PrEP), post-exposure prophylaxis (PEP), sexual history taking, HIV/STI testing, and others.

#### Project ECHO^®^-Standard (PE-S)

Participants engaged in bi-weekly 60-minute PE-S directed by hub members who are lead experts in infectious diseases, mental and behavioural health, and family medicine. The lead hub members facilitated the case presentation and discussions.

#### Project ECHO^®^ for Stigma Reduction (PE-SR)

Participants received the PE-SR training bi-weekly for 60 min. Lead hub members in this arm included infectious disease physicians, mental and behavioural health experts, family medicine specialists, stigma experts, and community key opinion leaders. Lead hub members facilitated the case presentation and discussions. The stigma reduction tools embedded into this study arm aimed to change social norms and behaviours, educate, and facilitate contact with key populations and PWH longitudinally over nine months consistently.

In summary, the following are distinct characteristics between the two Project ECHO^®^ arms: (1) Teaching modules: PE-SR included modules that were specifically targeted for affective and behavioural changes theoretically impacted by stigma, i.e., modules on mental and behavioural health, substance use and addiction, social determinants of health, in-depth didactics on key populations and intersectionality, stigma and healthcare, gender and sexuality, sexual and reproductive health, and stigma-reduction interventions in healthcare settings. (2) Hub members: PE-SR included expert panels from community members with lived experience, leading didactics and case discussions compared to PE-S. This allowed the stigma-enhanced model to move from a medicalised model to one constructed on a social model of health. (3) Enhanced content: Rooted in contact theory, guest speakers with lived experience (e.g., a PrEP user, a female sex worker, a transgender woman, a person in recovery from substance use, and a person living with schizophrenia) shared their lived experience, discussed how stigma has impacted them and how to reduce stigma. We presented findings from our previous work using Photovoice [39] in the form of videos during the sessions. We shared perspectives from community members who discussed their sources of happiness and sadness, challenges and stressors, personal priorities, and experiences with and hopes for doctor interactions.

### Participants

Using convenience sampling, we recruited general practitioners and family medicine specialists from the Family Medicine Specialists’ Association (FMSA), the Malaysian Medicine Association (MMA), the Malaysian Primary Care Network, and the Doctors Only Bulletin Board System (DOBBS). We defined general practitioners as clinicians who had completed their residency and practised at various public or private healthcare facilities throughout Malaysia. Family medicine specialists are primary care physicians who have completed their training in family medicine and are working at government and/or private primary care centres in Malaysia. We excluded physicians who were unable to commit to the scheduled bi-weekly training sessions.

From the total number of recruitments received via the study registration form, 78 clinicians were randomly selected using block randomisation with varying block lengths on R. We collected contact details from individuals who agreed to participate in the study. Clinician identities were confirmed by cross-referencing national registries, such as the Medical Register Information and Technical System (MeRITS). Subsequently, the study coordinators contacted the clinicians’ medical directors to describe the study and officially request permission to participate. The research team also requested that medical directors protect the participants’ time needed for the project, up to 3 h per month, for nine continuous months. Clinicians received Continuous Professional Development points and a RM30 (approximately USD 7) lunch voucher for each session attended, and an e-certificate upon completing the training. Participants who completed our surveys also received RM75 (approximately USD 17) per survey as a token of appreciation.

### Measures

We administered quantitative surveys through Qualtrics. At baseline, we collected sociodemographic (e.g., age, gender, faith affiliation, personal contact with key populations and people with HIV) and clinically relevant information (e.g., years of practising, clinical rank, working experience with key populations, HIV care provision and PrEP prescription). At midline and endline, we included the HIV activism measure. The measurement of HIV activism employed the HIV Activist Identity, Commitment, and Orientation Scale, which has been psychometrically evaluated to measure HIV activism among clinicians in Malaysia (Cronbach’s α of 0.91) [40]. We asked participants to rate their agreement with six statements reflecting their social collective identity and commitment to HIV activism based on a Likert-type rating system ranging from 1 (strongly disagree) to 5 (strongly agree). The HIV activism orientation constructs assessed clinicians’ engagement in individual and group-based activism from simple everyday involvement to structural activism, on a continuum from 1 (never) to 5 (often). We formed composite means for each construct.

### Analysis Plan

We employed an intent-to-treat approach to assess the effects of the intervention, wherein losses to follow-up were accounted for, and participants were analysed in the study groups in which they were randomised. Each study arm served as a predictor in which the clinicians were randomised. The study’s outcome is the change in HIV activist identity, commitment, and orientation over time across study arms.

We tested for significant differences in baseline socio-demographic and clinical characteristics using one-way analysis of variance (ANOVA) for continuous variables or Pearson’s chi-square test for categorical variables between the study groups to rule out differences that could threaten the study’s internal validity in exploring causal relationships between trial outcomes. Next, we evaluated the effects of any significant differences in baseline sociodemographic and clinical characteristics between study groups, predicting the changes in HIV activism scores using multivariable linear regression. Categorical sociodemographic and nominal clinical characteristics, i.e., variables that employ two or more categories, were dummy coded before incorporating the variables into the multivariable linear regression model [41].

To determine the effects of the stigma reduction tools embedded in the ECHO tele-training platform on HIV activism, we performed a 3 by 2 (3 conditions and 2 survey points) repeated-measure analysis of covariance (ANCOVA). This allowed us to examine significant differences over time between intervention groups and linear interactions with any sociodemographic and clinical characteristics (serving as covariates), shown in the build-up analyses with the changes in HIV activism scores. The repeated measure ANCOVA model sought to test, with the covariates, (1) within-group differences (time effect), (2) between-group differences (group effect), and (3) within-between-group differences (time-group interaction).

## Results

### Clinician Sociodemographic and Clinical Characteristics

All 78 participants were, on average, 37 years old, primarily identified as Chinese (44.9%) and ‘Other’ faith affiliation (47.4%), with over half (59%) identifying as women (see Appendix 2). Most participants were general practitioners or medical officers (47.4%) and were working in the government sector (74.4%). Participants had varying working experience and personal contact across key populations. Most clinicians had minimal contact with key populations or PWH. While many of them are proficient in performing HIV testing, most were not familiar with PrEP prescribing.

### Group Characteristics at the Start of the Trial

We tested the mean differences in sociodemographic and clinical characteristics of the clinicians at baseline with one-way ANOVA (see Appendix 2). There were statistically significant mean differences in two socio-demographic characteristics, i.e., ethnicity (χ²=11.14, *p*-value = 0.03) and faith affiliation (χ²=10.45, *p*-value = 0.03).

The multivariable linear regression models with all sociodemographic and clinical characteristics predicting changes in HIV activism scores (see Appendix 3) suggested that participants who had more contact with PWID were more likely to identify and commit to HIV activism (β = 0.35, 95% CI: 0.05, 0.48, *p*-value = 0.02). Younger participants had more experience practicing medicine and more contact with PWID and FSW were also likely to engage in day-to-day HIV activism (Age, β=-1.34, 95% CI: -0.32, -0.01, *p*-value = 0.04; Years practicing, β = 0.08, 95% CI: 0.02, 0.34, *p*-value = 0.03; contact with PWID, β = 0.37, 95% CI: 0.12, 0.68, *p*-value = 0.01). Lastly, older participants and participants who had more contact with TGW were more likely to engage in structural HIV activism (Age, β = 1.37, 95% CI: 0.01, 0.38, *p*-value = 0.04; contact with TGW, β = 0.31, 95% CI: 0.04, 0.62, *p*-value = 0.03). We subsequently controlled for these variables in the repeated-measure ANCOVA.

### Intention-to-Treat Repeated Measure ANCOVA

*Within-Group Difference (Time Effect).* As expected from our multivariable linear regression models, participants who had greater contact with PWID were more likely to identify and commit to HIV activism [F(1,65) = 3.99, η²_p_= 0.058, *p* < 0.05]. Similarly, we observed positive differences in orientation towards structural HIV activism after controlling for age, years practicing medicine and mean difference in contact with TGW [F(1,65) = 4.76, η²_p_=0.068 *p*-value < 0.05; F (1,65) = 6.78, η²_p_= 0.094, *p*-value < 0.05; F (1,65) = 6.33, η²_p_= 0.089 *p*-value < 0.05] (Table 1).

**Table 1:** Tests of within-group effects

Construct	Mean (SD)		F(df), η²_p_							
	T1	T2	Time	Time x Age	Time x Years Practising	Time x Contact				
						MSM	TGW	PWID	FSW	PWH
HIV Identity & Commitment	3.40 (0.73)	3.42 (0.75)	2.35 (1, 65), 0.035	2.58 (1, 65), 0.038	2.54 (1, 65), 0.038	0.25 (1, 65), 0.004	1.26 (1, 65), 0.019	3.99* **(1**,** 65)**,** 0.058**	0.18 (1, 65), 0.003	0.05 (1, 65), 0.001
Orientation Towards Day-to-day HIV Activism	2.94 (0.92)	2.97 (0.88)	3.42 (1, 65), 0.050	3.33 (1, 65), 0.049	2.70 (1, 65), 0.040	0.11 (1, 65), 0.737	0.14 (1, 65), 0.002	2.59 (1, 65), 0.038	0.07 (1, 65), 0.001	0.75 (1, 65), 0.011
Orientation Towards Structural HIV Activism	2.25 (0.96)	2.26 (0.95)	3.68 (1, 65), 0.054	4.76* **(1**,** 65)**,** 0.068**	6.78* **(1**,** 65)**,** 0.094**	1.09 (1, 65), 0.017	6.33* **(1**,** 65)**,** 0.089**	0.13 (1, 65), 0.002	0.18 (1, 65), 0.003	2.72 (1, 65), 0.040

Standard Deviations: SD, F−values (degrees of freedom): F(df), Partial eta−squared (η²ₚ), men who have sex with men (MSM), transgender women (TGW), people who inject drugs (PWID), female sex workers (FSW), people with HIV (PWH), Time Stamp 1 (T1), Time Stamp 2 (T2), *p<0.05

*Between-Group Difference (Group Effect).* We observed no significant differences between the three study groups across all HIV activism outcomes (see Table 2), even after controlling for age, years of practice, and contact with key populations.

**Table 2:** Tests of between-groups effects

Construct	Mean (SD)			F(df), η²_p_	Sig.
	PE-S	PE-SR	HC		
HIV Identity & Commitment	3.64 (0.83)	3.35 (0.64)	3.26 (0.71)	2.50 (2, 63), 0.074	> 0.05
Orientation Towards Day-to-day HIV Activism	3.23 (0.95)	2.87 (0.76)	2.78 (0.88)	2.000 (2, 63), 0.060	> 0.05
Orientation Towards Structural HIV Activism	2.26 (0.99)	2.53 (0.83)	1.99 (0.98)	2.396 (2, 63), 0.071	> 0.05

Standard Deviations: SD, F−values (degrees of freedom): F(df), Partial eta−squared (η²ₚ), Project ECHO: Standard (PE−S), Project ECHO: Stigma Reduction (PE−SR), HIV−Connect (HC), Significance/*p*−values (Sig.)

*Within-Between Groups Difference (Time-Group Interaction).* The changes in HIV activism scores at two levels of time were not significantly different between the study arms after controlling for the covariates

The pairwise comparison revealed that participants in PE-S reported greater HIV activist identity and commitment than participants in HC (Mean Difference = 0.38; 95% CI: 0.03, 0.74; *p*-value < 0.05). Interestingly, participants in PE-SR reported greater orientation towards structural HIV activism compared to the HC arm (Mean Difference = 0.54; 95% CI: 0.05, 1.02; *p*-value < 0.05) (Table 3).

**Table 3:** Tests of within-between-groups effects

Construct	Time	Mean (SD)			F(df), η²_p_	Sig.
		PE-S	PE-SR	HC		
HIV Identity & Commitment	T1	3.58 (0.87)	3.31 (0.58)	3.33 (0.73)	1.61 (2, 63), 0.049	> 0.05
	T2	3.72 (0.77)	3.35 (0.71)	3.21 (0.70)		
	T2-T1	0.14	0.04	-0.12		
Orientation Towards Day-to-day HIV Activism	T1	3.20 (1.01)	2.89 (0.73)	2.74 (0.97)	0.25 (2, 63), 0.008	> 0.05
	T2	3.25 (0.98)	2.85 (0.80)	2.82 (0.81)		
	T2-T1	0.05	-0.04	0.08		
Orientation Towards Structural HIV Activism	T1	2.15 (0.90)	2.54 (0.93)	2.08 (1.01)	0.87 (2, 63), 0.027	> 0.05
	T2	2.31 (1.07)	2.54 (0.74)	1.94 (0.96)		
	T2-T1	0.16	0	-0.14		

Standard Deviations: SD, F−values (degrees of freedom): F(df), Partial eta−squared (η²ₚ), Time Stamp 1 (T1), Time Stamp 2 (T2), Project ECHO: Standard (PE−S), Project ECHO: Stigma Reduction (PE−SR), HIV−Connect (HC), Significance/*p*−values (Sig.)

## Discussion

In this study, we piloted a tele-training program targeted at primary care physicians and general practitioners and evaluated participants’ HIV activist identity, commitment, and orientation towards day-to-day and structural HIV activism. We compared the intervention arm with the inclusion of stigma reduction tools (PE-SR) with two comparator groups: an arm that employed the Project ECHO^®^ model without the inclusion of stigma reduction tools (PE-S), and a treatment-as-usual arm, HIV Connect (HC), an existing HIV Prevention training module for clinicians.

The repeated-measure ANCOVA analyses yielded mixed results. When covariates were controlled, there were significant improvements in HIV activist identity and commitment and orientation towards structural HIV activism emerging from all groups across time. We also observed an improved orientation towards structural HIV activism in association with contact with TGW among participants in PE-SR.

The variables (age, years of clinical experience, and contact with key populations) were controlled in the analyses. N. M. Hed and Grasson [42], in their work investigating the differences in political engagement across age groups in Malaysia, found a significant age-related pattern in individuals’ involvement in political activism. They found that younger individuals in Malaysia are less likely to engage politically than older individuals. In the study conducted by Yankah and colleagues [43], regression models also found chronological age to be a significant predictor of conventional and high-risk activism; older participants were also found to engage more in activism. Engagement in structural HIV activism among senior clinicians could be attributed to their role in senior management, and they tend to be more influential than younger clinicians. Livingston and colleagues [44], in their systematic review of stigma reduction interventions, revealed that contact (particularly in the form of positive storytelling with people with substance use disorder) is an effective strategy to reduce stigma. Several studies demonstrated that contact with people with substance use disorders resulted in improved comfort in discussing and taking clinical history, attitudes towards pregnant women with substance use disorders, and reduction in negative thoughts and stigmatizing attitudes towards people with substance use disorders, especially people with heroin and alcohol use dependence [45, 46, 47].

PE-SR overall showed a statistically significant positive difference in orientation towards structural activism. Although within-group analyses showed that the arm did not yield a positive and significant mean difference, there are a few reasons it still emerged as a group with a statistical difference compared to the other comparator groups. Firstly, because there is no difference in mean scores from T1 and T2, it could suggest that the group has reached a ceiling effect, and any further intervention would not have improved it further. Secondly, improvements in other comparator arms were not significant enough to outperform PE-SR. Nyblade and colleagues [48], in their systematic review of stigma reduction interventions for healthcare facilities, showed that of the 40 unique quantitative studies, 13 had mixed results. While these findings suggest that more randomised controlled trials are needed, it was not too surprising that our study yielded mixed results.

### Limitations and Future Directions

It is also essential to consider the limitations of the study. First, the sampling technique employed in the study may have introduced selection bias or raised the risk of information contamination across study arms through informal communications due to overlapping working clinical settings or shared professional networks. Although we believe these biases have been minimised via the structured delivery, blinding, and recruitment of participants across Malaysia, future studies should adopt other recruitment strategies and explore possible associations between participants. Additionally, the study included a relatively small sample of clinicians. Although the sample size yielded sufficient power with a medium effect size, we may be able to observe stronger significant effects with a larger sample size in the performed analyses, and our results may be more generalizable. The relatively small sample may also raise the risk of violating assumptions of normality and homogeneity of variance required for repeated-measure ANCOVA. While we employed repeated measures ANCOVA to examine group differences over time, we acknowledge that linear mixed-effects models (LMMs) offer greater flexibility in handling correlated data, small samples, and missingness. In this study, our data were largely complete with a balanced design, and the assumptions for repeated measures ANCOVA were adequately met. Given these conditions and our aim to compare mean differences across time points while adjusting for covariates, we deemed repeated measures ANCOVA appropriate. Nonetheless, we recognise the advantages of LMMs and recommend their use in future studies, particularly when data are unbalanced or missingness is more pronounced. Thirdly, responses to the survey are self-reported and may not truly represent the sample’s engagement in activism, prejudices, stereotypes, and their endorsement of discrimination. Recall bias and social desirability may influence the responses [49]. Furthermore, because participants voluntarily participated in the training (or the study), their attitudes towards key populations and PWH were likely not extreme. Clinicians with the most negative views or who do not endorse HIV activism might be less inclined to volunteer for training programs aimed at tackling gaps in the HIV response. Lastly, the measures of HIV activism in the trial were taken twice. Measuring activism more frequently, including a minimum of one month post-training, could give us the advantage of robust analyses of the changes in HIV activism, healthcare service outcomes, and stigma overall, including stability of the effects post-intervention.

While the study holds reasonable preliminary findings, future research on expanding the understanding of HIV activism can benefit from methodological improvements. Future research should test how HIV activism impacts HIV screening, uptake of PrEP, linkage of care, and ART prescription. This allows us to tangibly understand how HIV activism impacts the society enablers of the HIV response, i.e., the 95-95-95 goals. Future research could also utilise other randomisation methods, e.g., cluster randomisation based on clinicians in healthcare facilities. Not only does this allow the intervention to be focused while evaluating the impact of the stigma reduction intervention on a single healthcare facility instead of individual clinicians, but it may be a more effective form of social norming within the facility. It may allow researchers to assess structural activism. Future studies could also evaluate the impact of HIV activism with similar allocation of groups but employ other forms of trials (e.g., stepped wedge randomised controlled trial). This may allow researchers to conduct dose-response analyses and test the efficacy of each stigma reduction tool on HIV activism. With a larger sample size, studies can also evaluate intervention modifiers (e.g., teaching effectiveness, acceptability, appropriateness, and feasibility, or session attendance). Lastly, future researchers can explore the stability of treatment effects using longitudinal studies or evaluate the changes in HIV across at least three time points.

## Conclusion

The randomised controlled trial yielded mixed results. Notwithstanding the study’s limitations, the Project ECHO^®^ training model, with the inclusion of stigma reduction tools, suggests preliminary improvement in HIV activism, particularly in orientation towards structural HIV activism. Findings suggest that clinicians can be empowered to drive tangible change, hand-in-hand with communities living with and affected by HIV

## Electronic Supplementary Material

Below is the link to the electronic supplementary material.

Supplementary Material 1

## Data Availability

Data may be available from the corresponding author upon request.
