# Supplementary material for: Strengthening HIV Activism Among Clinicians in Malaysia: A Randomised Controlled Trial
Source: AIDS Behav. 2025 Jul 21;29(12):3931–9. doi: 10.1007/s10461-025-04829-1 (PMC12580431; doi:10.1007/s10461-025-04829-1)
Supplement: Supplementary file 1 — Supplementary Material 1 [file 10461_2025_4829_MOESM1_ESM.docx]

# **Electronic Supplementary Material**

## Appendix 1: CONSORT Flow Diagram


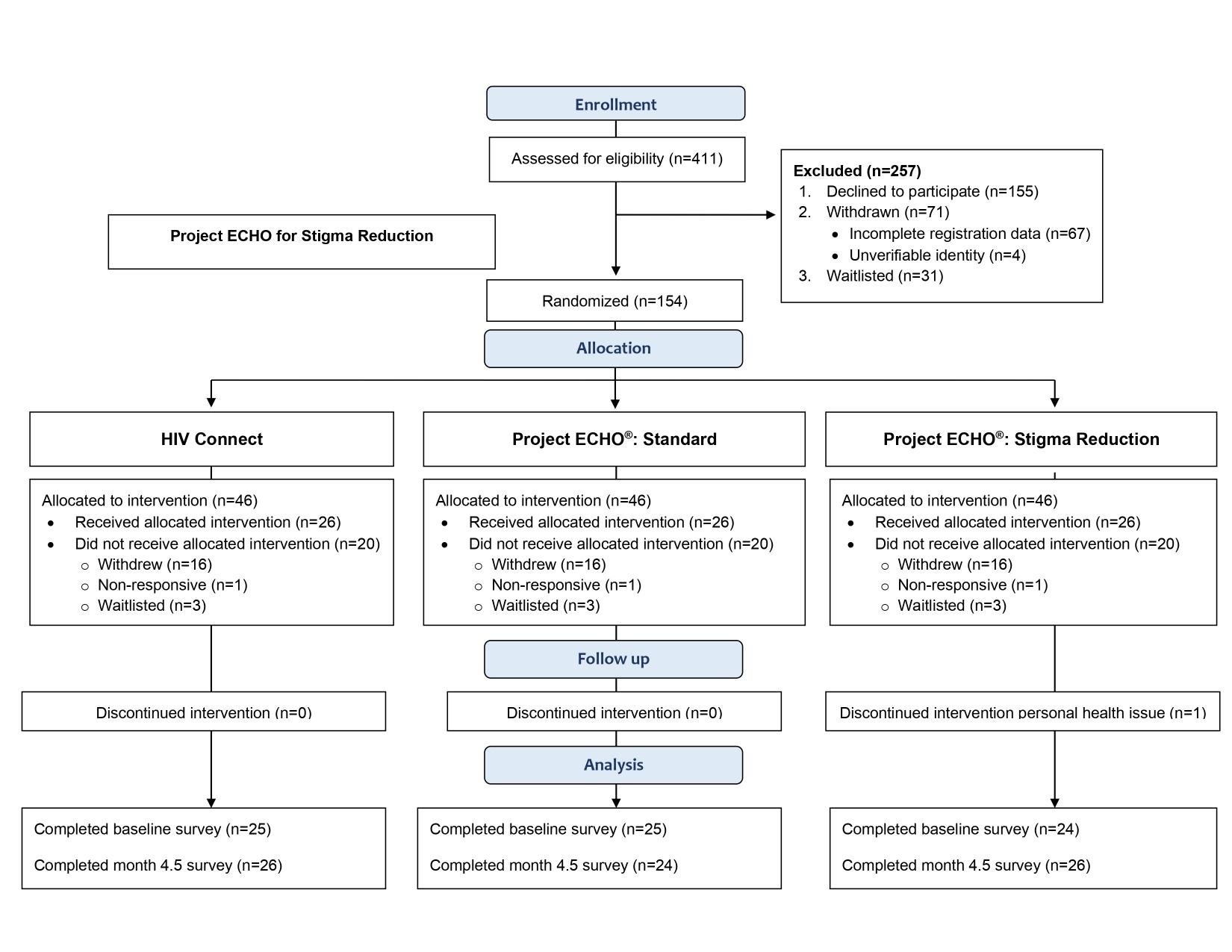


## Appendix 2: Mean differences in sociodemographic and clinical characteristics of the clinicians across study arms

| Characteristics | | n (%) or Mean (SD) | | | | F or χ² | p-values |
| --- | --- | --- | --- | --- | --- | --- | --- |
|  |  | Overall (n=78) | Study arms | | |  |  |
|  |  |  | HC (n=26) | PE-S (n=26) | PE-SR (n=26) |  |  |
| **Sociodemographic** | | | | | | | |
| Age | | 37.35 (6.05) | 37.42(6.37) | 36.50(4.05) | 38.23(7.28) | 0.53 | 0.59 |
| Gender | Man | 32 (41.03) | 13 (50.00) | 9 (34.62) | 10 (38.46) | 1.37 | 0.52 |
|  | Women | 46 (58.97) | 13 (50.00) | 17 (65.38) | 16 (61.54) |  |  |
| Ethnicity | Malay | 28 (35.90) | 4 (15.38) | 14 (53.85) | 10 (38.46) | **11.14** | **0.03** |
|  | Chinese | 35 (44.87) | 16 (61.54) | 6 (23.08) | 13 (50.00) |  |  |
|  | Indian and others | 15 (19.23) | 6 (23.08) | 6 (23.08) | 3 (11.54) |  |  |
| Faith affiliation | Muslim | 28 (35.90) | 4 (15.38) | 14 (53.85) | 10 (38.46) | **10.45** | **0.03** |
|  | Christian | 13 (16.67) | 4 (15.38) | 3 (11.54) | 6 (23.08) |  |  |
|  | Others | 37 (47.44) | 18 (69.23) | 9 (34.62) | 10 (38.46) |  |  |
| Previous Contact in Personal Life | |  | | | | | |
| FSW | No | 68 (87.18) | 23 (88.46) | 22 (84.62) | 23 (88.46) | 2.70 | 0.61 |
|  | Acquaintance | 9 (11.54) | 3 (11.54) | 4 (15.38) | 2 (7.69) |  |  |
|  | Close friends or family | 1 (1.28) | 0 (0) | 0 (0) | 1 (3.85) |  |  |
| PWID | No | 55 (70.51) | 19 (73.08) | 16 (61.54) | 20 (76.9) | 2.33 | 0.68 |
|  | Acquaintance | 21 (26.92) | 6 (23.08) | 9 (34.62) | 6 (23.08) |  |  |
|  | Close friends or family | 2 (2.56) | 1 (3.85) | 1 (3.85) | 0 (0) |  |  |
| TGW | No | 52 (66.67) | 17 (65.38) | 16 (61.54) | 19 (73.08) | 1.52 | 0.82 |
|  | Acquaintance | 24 (30.77) | 8 (30.80) | 9 (34.60) | 7 (26.92) |  |  |
|  | Close friends or family | 2 (2.56) | 1 (3.85) | 1 (3.85) | 0 (0) |  |  |
| MSM | No | 33 (42.31) | 11 (42.31) | 8 (30.77) | 14 (53.80) | 4.64 | 0.33 |
|  | Acquaintance | 30 (38.46) | 8 (30.80) | 13 (50.00) | 9 (34.62) |  |  |
|  | Close friends or family | 15 (19.23) | 7 (26.90) | 5 (19.23) | 3 (11.54) |  |  |
| PWH | No | 41 (52.56) | 15 (57.69) | 13 (50.00) | 13 (50.00) | 2.93 | 0.57 |
|  | Acquaintance | 26 (33.33) | 6 (23.08) | 9 (34.60) | 11 (42.31) |  |  |
|  | Close friends or family | 11 (14.10) | 5 (19.23) | 4 (15.38) | 2 (7.69) |  |  |
| **Clinical** | | | | | | | |
| Affiliation | Government sector | 58 (74.36) | 19 (73.08) | 19 (73.08) | 20 (76.92) | 0.44 | 0.80 |
|  | Private Sector | 20 (25.64) | 7 (26.90) | 7 (26.92) | 6 (23.08) |  |  |
| Years Practicing Medicine | | 11.78 (6.16) | 11.77 (5.80) | 10.96 (4.31) | 12.77 (7.89) | 0.56 | 0.57 |
| Clinical rank | Medical Officer | 20 (25.64) | 7 (26.92) | 7 (26.92) | 6 (23.10) | 1.27 | 0.87 |
|  | Registrar, Specialist, or Consultant | 41 (52.56) | 12 (46.15) | 15 (57.69) | 14 (53.80) |  |  |
|  | General Practitioner | 17 (21.79) | 7 (26.92) | 4 (15.38) | 6 (23.08) |  |  |
| Contact with key populations | |  | | | | | |
| FSW | Not at all | 22 (28.21) | 6 (23.08) | 7 (26.92) | 9 (34.62) | 3.43 | 0.75 |
|  | Very little | 34 (43.59) | 11 (42.31) | 11 (42.31) | 12 (46.15) |  |  |
|  | Somewhat | 19 (24.36) | 8 (30.77) | 6 (23.08) | 5 (19.23) |  |  |
|  | A great deal | 3 (3.85) | 1 (3.80) | 2 (7.69) | 0 (0) |  |  |
| PWID | Not at all | 5 (6.41) | 3 (11.50) | 1 (3.80) | 1 (3.80) | 3.36 | 0.76 |
|  | Very little | 26 (33.33) | 8 (30.77) | 10 (38.46) | 8 (30.77) |  |  |
|  | Somewhat | 26 (33.33) | 10 (38.46) | 8 (30.77) | 8 (30.77) |  |  |
|  | A great deal | 21(26.92) | 5 (19.23) | 7 (26.92) | 9 (34.62) |  |  |
| TGW | Not at all | 17 (21.79) | 5 (19.23) | 7 (26.92) | 5 (19.23) | 7.58 | 0.27 |
|  | Very little | 33 (42.31) | 9 (34.62) | 8 (30.77) | 16 (61.54) |  |  |
|  | Somewhat | 20 (25.64) | 9 (34.62) | 7 (26.92) | 4 (15.38) |  |  |
|  | A great deal | 8 (10.26) | 3 (11.50) | 4 (15.38) | 1 (3.80) |  |  |
| MSM | Not at all | 3 (3.85) | 2 (7.69) | 1 (3.80) | 0 (0) | 4.05 | 0.67 |
|  | Very little | 13 (16.67) | 4 (15.38) | 5 (19.23) | 4 (15.38) |  |  |
|  | Somewhat | 26 (33.33) | 6 (23.08) | 10 (38.46) | 10 (38.46) |  |  |
|  | A great deal | 36 (46.15) | 14 (53.80) | 10 (38.46) | 12 (46.15) |  |  |
| PWH | Not at all | 3 (3.85) | 2 (7.69) | 0 (0) | 1 (3.80) | 8.12 | 0.23 |
|  | Very little | 14 (17.95) | 4 (15.38) | 8 (30.77) | 2 (7.69) |  |  |
|  | Somewhat | 24 (30.77) | 6 (23.08) | 7 (26.92) | 11 (42.31) |  |  |
|  | A great deal | 37 (47.44) | 14 (53.80) | 11 (42.31) | 12 (46.15) |  |  |
| HIV Testing Experience | Novice | 3 (3.85) | 1 (3.80) | 0 (0) | 2 (7.69) | 3.74 | 0.44 |
|  | Proficient | 47 (60.26) | 18 (69.23) | 16 (61.54) | 13 (50.00) |  |  |
|  | Experienced | 28 (35.90) | 7 (26.92) | 10 (38.46) | 11 (42.31) |  |  |
| PrEP Prescribing Experience | Novice | 46 (58.97) | 11 (42.31) | 18 (69.23) | 17 (65.38) | 6.51 | 0.16 |
|  | Proficient | 28 (35.90) | 12 (46.15) | 8 (30.77) | 8 (30.77) |  |  |
|  | Experienced | 4 (5.13) | 3 (11.50) | 0 (0) | 1 (3.80) |  |  |

^Note: Differences between study groups were tested using one-way analysis of variance (ANOVA) for continuous variables or Pearson’s chi-square test for categorical variables, female sex worker (FSW), people who inject drugs (PWID), transgender women (TGW), men who have sex with men (MSM), people with HIV (PWH)^

## Appendix 3: Association between sociodemographic and clinical characteristics with the changes in HIV activism scores

|  | | **HIV Identity and Commitment** | | | | | **Orientation Towards Day-to-day HIV activism** | | | | | **Orientation Towards Structural HIV activism** | | | | |
| --- | --- | --- | --- | --- | --- | --- | --- | --- | --- | --- | --- | --- | --- | --- | --- | --- |
|  | | **β** | **SE** | **Sig** | **95% CI** | | **β** | **SE** | **Sig** | **95% CI** | | **β** | **SE** | **Sig** | **95% CI** | |
|  |  |  |  |  | **LB** | **UB** |  |  |  | **LB** | **UB** |  |  |  | **LB** | **UB** |
| Age | | 0.11 | 0.06 | 0.87 | -0.11 | 0.13 | **-1.34** | **0.08** | **0.04** | **-0.32** | **-0.01** | **1.37** | **0.09** | **0.04** | **0.01** | **0.38** |
| Gender | | -0.22 | 0.16 | 0.19 | -0.60 | 0.12 | -0.19 | 0.24 | 0.22 | -0.77 | 0.18 | 0.12 | 0.28 | 0.46 | -0.35 | 0.77 |
| Ethnicity | Malay | Ref. | | | | | | | | | | | | | | |
|  | Chinese | 0.06 | 0.14 | 0.67 | -0.22 | 0.34 | 0.25 | 0.20 | 0.22 | -0.15 | 0.66 | -0.16 | 0.23 | 0.49 | -0.62 | 0.30 |
|  | Indian and Others | -0.11 | 0.18 | 0.55 | -0.48 | 0.26 | 0.09 | 0.26 | 0.74 | -0.44 | 0.62 | 0.13 | 0.30 | 0.66 | -0.47 | 0.73 |
| Faith affiliation | Muslim | Ref. | | | | | | | | | | | | | | |
|  | Christian | 0.00 | 0.19 | 0.99 | -0.38 | 0.38 | 0.39 | 0.27 | 0.16 | -0.15 | 0.93 | -0.27 | 0.31 | 0.39 | -0.88 | 0.35 |
|  | Others | -0.02 | 0.14 | 0.87 | -0.30 | 0.26 | 0.12 | 0.20 | 0.57 | -0.28 | 0.52 | -0.10 | 0.23 | 0.66 | -0.56 | 0.36 |
| Year Practicing | | -0.29 | 0.06 | 0.69 | -0.15 | 0.10 | **0.08** | **0.18** | **0.03** | **0.02** | **0.34** | -0.18 | 0.10 | -1.90 | 0.06 | -0.37 |
| Contact with key populations | FSW | 0.03 | 0.12 | 0.84 | -0.22 | 0.27 | 0.12 | 0.16 | 0.45 | -0.20 | 0.44 | -0.10 | 0.19 | 0.52 | -0.50 | 0.26 |
|  | PWID | **0.35** | **0.11** | **0.02** | **0.05** | **0.48** | **0.37** | **0.14** | **0.01** | **0.12** | **0.68** | 0.02 | 0.16 | 0.86 | -0.30 | 0.36 |
|  | TGW | 0.01 | 0.09 | 0.96 | -0.18 | 0.19 | 0.08 | 0.12 | 0.57 | -0.18 | 0.32 | **0.31** | **0.14** | **0.03** | **0.04** | **0.62** |
|  | MSM | 0.02 | 0.11 | 0.90 | -0.21 | 0.23 | -0.12 | 0.14 | 0.38 | -0.42 | 0.16 | -0.15 | 0.17 | 0.28 | -0.53 | 0.15 |
|  | PWH | 0.05 | 0.13 | 0.71 | -0.22 | 0.32 | 0.12 | 0.17 | 0.36 | -0.19 | 0.51 | 0.20 | 0.21 | 0.13 | -0.10 | 0.73 |

^Abbreviations: Standardized Beta (β), Standard Error (SE), Significance/p-values (Sig.), 95% confidence interval (95% CI), Lower bound (LB), Upper bound (UB), female sex worker (FSW), people who inject drugs (PWID), transgender women (TGW), men who have sex with men (MSM), people with HIV (PWH)^
